# Anti-Tick-Bourne Encephalitis IgM Intrathecal Synthesis as a Prediction Marker in Tick-Borne Encephalitis Patients

**DOI:** 10.3390/microorganisms13010213

**Published:** 2025-01-20

**Authors:** Piotr Czupryna, Sambor Grygorczuk, Agnieszka Siemieniako-Werszko, Jakub Okrzeja, Justyna Dunaj-Małyszko, Justyna Adamczuk, Sławomir Pancewicz, Joanna Zajkowska, Karolina Narejko, Joanna Oklińska, Gabriela Trojan, Anna Moniuszko-Malinowska

**Affiliations:** Department of Infectious Diseases and Neuroinfections, Medical University of Bialystok, 15-540 Bialystok, Poland; avalon-5@wp.pl (P.C.); sambor.grygorczuk@umb.edu.pl (S.G.); neuroin@umb.edu.pl (A.S.-W.); jakubokrzeja086@gmail.com (J.O.); justyna.dunaj-malyszko@umb.edu.pl (J.D.-M.); justyna.adamczuk11@gmail.com (J.A.); slawomir.pancewicz@umb.edu.pl (S.P.); joanna.zajkowska@umb.edu.pl (J.Z.); karolina.narejko99@gmail.com (K.N.); joanna.oklinska@gmail.com (J.O.); gabrielaatrojan@gmail.com (G.T.)

**Keywords:** tick-borne encephalitis, TBE, intrathecal synthesis, IgM, CSF

## Abstract

The aim of this study was to evaluate the usefulness of IgM anti-Tick-Borne Encephalitis (anti-TBE) intrathecal synthesis in the diagnosis and prediction of the clinical course of the disease. Thirty-six patients were included in the study (patients reported symptoms such as fever, headache, fatigue, and nausea/vomiting). CRP, White Blood Cells (WBC), pleocytosis, Cerebrospinal Fluid (CSF) protein concentration, CSF albumin concentration, serum IgM, serum IgG, CSF IgM, CSF IgG, IgM Index, IgG Index, and IgG Index/IgM Index ratio were the parameters which were examined in the individuals. An analysis of correlation presented statistical significance between IgM Index and pleocytosis and protein concentration in CSF in the whole group of individuals. IgM Index and IgG Index/IgM Index ratio may be used in the prediction of severity of TBE. The most probable link between the IgM intrathecal production and severity of TBE may be a result of delayed seroconversion to IgG, and therefore not an adequate response to the virus presence.

## 1. Introduction

Tick-borne encephalitis (TBE) is a viral infectious disease that affects the central nervous system (CNS) and is caused by the tick-borne encephalitis virus (TBEV), a member of the *Flavivirus* genus within the *Flaviviridae* family [1,2]. The reservoir of TBE virus is mainly made up of small rodents (voles, mice), but also includes insectivores and carnivores. Indicator hosts supporting virus circulation indirectly by enabling tick multiplication include different species of wild and domestic mammals (e.g., foxes, bats, hares, deer, wild boar, sheep, cattle, goats, and dogs) [3]. TBEV is primarily transmitted to humans through the bite of infected *Ixodes* ticks, which are the main vectors responsible for its spread. Additionally, although less common, TBEV infection can occur through the consumption of unpasteurized milk or dairy products derived from infected animals, such as goats, sheep, or cows.

The disease is endemic across wide geographical regions, particularly in northern, central, and eastern Europe, as well as parts of Asia, including Russia, China, and Japan [4,5]. These areas are characterized by suitable climates and habitats for tick populations, such as forests, grasslands, and rural environments. The risk of infection is highest during the warmer months, typically from spring to early autumn, when ticks are most active.

In recent years, the incidence of tick-borne encephalitis has been rising, which may be attributed to factors such as changing climatic conditions, increased outdoor recreational activities, and growing human encroachment into tick-infested habitats [6]. According to surveillance data, in 2020 alone, 24 European countries reported a total of 3817 cases of TBE, highlighting its significant public health impact [1]. In 2022, 20 European Union/European Economic Area (EU/EEA) countries reported a total of 3650 cases of tick-borne encephalitis (TBE), of which 3516 (96.3%) were confirmed.

The notification rate for the EU/EEA in 2022 was 0.81 per 100,000 population, representing a 14% increase compared to 2021. Eight countries reported no cases (Bulgaria, Iceland, Ireland, Liechtenstein, Luxembourg, Malta, Romania, and Spain). The highest numbers of confirmed cases in 2022 were reported by Czechia (*n* = 709), Germany (*n* = 554), and Sweden (*n* = 465) [7].

In Poland, 659 confirmed cases of TBE were reported in 2023 [8].

The diagnosis of tick-borne encephalitis (TBE) is established based on a combination of clinical manifestations and laboratory findings. A definitive diagnosis requires the presence of characteristic clinical symptoms, evidence of cerebrospinal fluid (CSF) pleocytosis, defined as a white blood cell count exceeding 5 × 10^6^ cells per litre, and the detection of specific antibodies against the tick-borne encephalitis virus (TBEV). These include the presence of anti-TBEV IgM and IgG immunoglobulins in the serum or, alternatively, the demonstration of TBEV IgG seroconversion over time [9].

The clinical signs associated with TBE encompass a spectrum of central nervous system (CNS) inflammation, and include meningitis, meningoencephalitis, and meningoencephalomyelitis [9]. Each of these forms represents varying degrees of severity and involvement of the CNS, from inflammation restricted to the meninges to more extensive damage involving the brain parenchyma and spinal cord.

TBEV-specific polymerase chain reaction (PCR) testing of blood is an additional diagnostic tool that can detect the presence of viral RNA, but its utility is limited to the early viremic phase of infection, which occurs before the onset of neurological symptoms. Once the disease progresses to the second phase, characterized by CNS inflammation, PCR testing is generally not effective due to the absence of detectable viremia at this stage [9].

In cases where the diagnosis remains uncertain or other conditions need to be ruled out, imaging studies of the brain and spinal cord, such as magnetic resonance imaging (MRI) or computed tomography (CT), may be useful. These imaging modalities can help identify structural or inflammatory changes consistent with TBE or exclude other potential causes of the patient’s symptoms, such as malignancies, demyelinating diseases, or other infections affecting the CNS [9]. Radiological studies in TBE patients reveal abnormalities involving the meninges, which may be more pronounced in the cerebellar folia and basal cisterns. Additionally, focal or diffuse hyperintensities are observed in the thalami, basal ganglia, cerebellum, and anterior horns of the spinal cord. However, these abnormalities are non-specific and transient [10].

There is a lack of clear data about markers that could help predict the course of TBE and potential sequelae development [11]. It has been proven that an intrathecal synthesis index of antibodies may be useful in the diagnosis of infectious diseases, such as syphilis, or in the prediction of the severity of various CNS diseases, including outcomes such as relapses and poor long-term outcomes in the course of anti-NMDA receptor (NMDAR) encephalitis [11,12].

Our previous study on the intrathecal synthesis of anti-tick-borne encephalitis (anti-TBE) antibodies showed that patients with TBE who have not fully recovered had significantly lower IgG intrathecal index at admission. Additionally, IgG2/IgG1 was significantly higher in patients who developed sequelae [13,14].

The aim of the study was to assess the usefulness of IgM anti-TBE intrathecal synthesis in the diagnosis and prediction of clinical course of the disease.

## 2. Materials and Methods

### 2.1. Clinical Criteria

This study included a total of 36 patients, comprising 10 females and 26 males, who were hospitalized in the Department of Infectious Diseases and Neuroinfections at the Medical University of Bialystok, Poland. The average age of the patients was 44.5 years, with the youngest being 22 years old and the oldest 76 years old, reflecting a broad age range of individuals affected by tick-borne encephalitis (TBE).

At the time of hospital admission, serum and cerebrospinal fluid (CSF) samples were collected from all patients. This timing corresponds to the onset of the second phase of TBE, which is characterized by neurological involvement and the appearance of symptoms such as headache, fever, and signs of central nervous system (CNS) inflammation. The decision to collect samples at this stage was critical for both diagnostic and research purposes, as it allowed for the identification of immunological and laboratory markers associated with the progression of the disease.

By focusing on patients during this specific phase of TBE, the study was able to examine the clinical and immunological characteristics of the disease when CNS involvement is most prominent. This approach ensured that the data collected would be relevant for understanding the diagnostic markers, severity, and progression of TBE, as well as for evaluating the efficacy of laboratory tests in confirming the diagnosis.

Patients complained about various symptoms, as presented in Table 1. Laboratory parameters such as data from cerebrospinal fluid (CSF) and serum are presented in Table 2.

None of the patients were vaccinated against TBE. The patients enrolled in the study fulfilled clinical, laboratory, and epidemiological criteria of the TBE definition.

The clinical criteria for a diagnosis of TBE include the presence of symptoms suggesting inflammation of the central nervous system (CNS), such as meningitis, meningoencephalitis, encephalomyelitis, or encephaloradiculitis. Meningitis was diagnosed based on inflammatory parameters in the cerebrospinal fluid (CSF), including elevated white blood cell count and protein concentration, in the absence of focal neurological symptoms. Accurate diagnosis relies on a combination of clinical assessment and laboratory findings to differentiate between various forms of CNS inflammation.

Meningoencephalitis was diagnosed when there were inflammatory parameters in CSF, altered consciousness, and the presence of focal neurological symptoms. Meningoencephalomyelitis was diagnosed when apart from meningoencephalitis symptoms, flaccid paralyses of the limbs were also present.

Among the analyzed patients, 18 were diagnosed with meningitis, while another 18 were diagnosed with meningoencephalitis. None of the patients, however, showed signs of meningoencephalomyelitis.

In 17 patients, sequelae were diagnosed at the time of discharge (the most common was cerebellar syndrome—9 patients).

All patients included in the study were local residents who did not report any recent travel abroad. This information is particularly relevant as it eliminates the possibility of exposure to *Flaviviridae* viruses other than the tick-borne encephalitis virus (TBEV) that may occur in other regions. Consequently, there was no necessity to exclude cross-reactive immune responses with other *Flaviviridae* viruses, such as West Nile virus (WNV) or yellow fever virus, which are not endemic to Poland. WNV has been detected exclusively in migratory birds for over 10 years, and despite active surveillance in humans, no confirmed cases have been reported to date [15]. WNV RNA detection is included in blood donor screening programmes, but no positive results have been identified thus far. In Poland, TBE remains the only flavivirus known to cause neuroinfection. Consequently, TBE is diagnosed based on positive serology, in accordance with the established case definition [16].

To date, there have been no confirmed cases of human infection with West Nile virus or yellow fever virus reported within Poland. The localized nature of the patient cohort and the epidemiological profile of Poland reinforce the specificity of the diagnosis and highlight the importance of geographical context in interpreting flaviviral infections.

TBE antibody titers in serum and CSF samples were measured with an Anti-TBEV ELISA (IgM, IgG) EUROIMMUN (Lubeck, Germany) test.

Serum samples were diluted at 1:404 (in accordance with the manufacturer’s recommendations) and CSF 1:2 volume ratio with a diluent provided by manufacturer. Intrathecal production of specific immunoglobulins was measured by relative CSF/Serum Quotient—CSQrel; it is the proportion of the quotient of specific immunoglobulins in a particular class (IgM or IgG) and the whole presence of IgM/IgG in CSF to the quotient of specific immunoglobulins in a particular class (IgM or IgG) and the whole presence of IgM/IgG in serum. A CSQrel value more than 1.5 indicates intrathecal specific anti-TBEV antibody production, a value of 1.3–1.5 means border results, and values 0.6–1.3 mean physiological norm, with no intrathecal antibody production, and values less than 0.6 mean unreliable results, according to the manufacturer’s instructions.

The IgG Index/IgM Index ratio was calculated. The IgG Index/IgM Index ratio was calculated to evaluate the balance between IgM and IgG responses. A prolonged nonspecific IgM response combined with a delayed IgG response may be associated with a more severe course of the disease.

Statistical analysis was performed using Statistica 12. Groups were compared by Mann–Whitney U test. ROC curve analysis was performed for potential biomarkers.

The study was approved by the National Science Centre (Poland) and the Bioethics Committee. Informed consent was obtained from the patients.

### 2.2. Ethical Issues

All methods were carried out in accordance with relevant guidelines and regulations of the National Science Centre (Poland) and the Bioethics Committee.

### 2.3. Data Availability

Data are available at NSC under project ID 2018/31/B/NZ6/02744, in detail, while well-established methods can be briefly described and appropriately cited.

In Table 3, specific antibody titers in serum and CSF of patients with meningitis and meningoencephalitis are presented. Additionally, the intrathecal synthesis index is showed.

**Table 3:** Specific antibody titers in serum and CSF of patients with meningitis and meningoencephalitis.

Meningitis *n* = 18						Meningoencephalitis *n* = 18			
TBE-Specific Antibodies									
	Median	Min	Max	Q1–Q3	Median	min	Max	Q1–Q3	*p*
serum IgM	116.3	53.3	250.2	74.8–222.8	153.3	26.4	219.3	84.8–184.8	0.78
serum IgG	559	159.5	2378.9	536.7–1562	511.7	113.9	1342.5	491.8–803	0.4
CSF IgM	39.9	13.1	190,2	19.2–96.1	93.25	16.2	190.2	28–150.2	0.06
CSF IgG	392.6	108.6	1746.4	258.1–698.2	795.8	116.9	4943.8	374.9–1212.5	0.05
Intrathecal synthesis									
	median	min	max	Q1–Q3	median	min	max	Q1–Q3	*p*
IgM Index	0.414	0.1	1.42	0.23–0.83	0.89	0.32	6.19	0.49–1.17	0.027
IgG Index	1.92	0.05	51.35	1.09–11.6	2.3	0.06	7.74	0.9–4.11	0.5
IgG Index/IgM Index ratio	7.84	0.58	62.12	3.77–13.49	2.19	0.08	16.1	0.77–3.17	0.003

**Figure 1:**
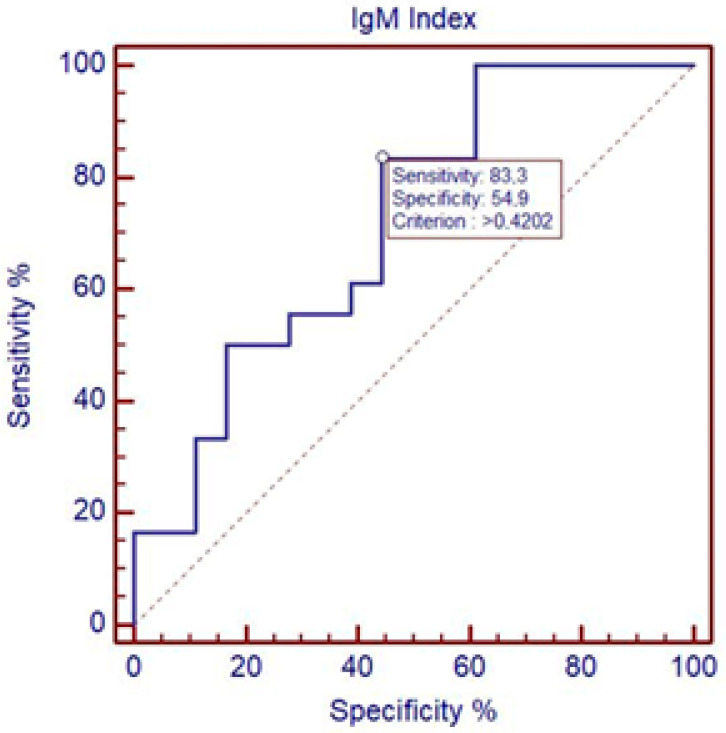
Differentiation between severe and mild course of TBE using IgM Index. ROC curve analysis showing that IgM Index differentiates between severe and mild courses of TBE at the cut-off 0.4202; specificity was 54.9% and sensitivity was 83.3%. AUC = 0.716, *p* < 0.05. CI (AUC) = 0.542 to 0.853.

Receiver operating characteristic curve (ROC curve) analysis showed that IgM Index differentiates between severe and mild courses of TBE at the cut-off at 0.4202; specificity was 54.9% and sensitivity was 83.3%. AUC = 0.716, *p* < 0.05 (Figure 1).

The IgM Index was significantly higher in TBE patients with a more severe course. The IgG/IgM Index was significantly lower in the severe TBE group. No differences between the two groups were observed in the case of serum or CSF antibody production.

**Figure 2:**
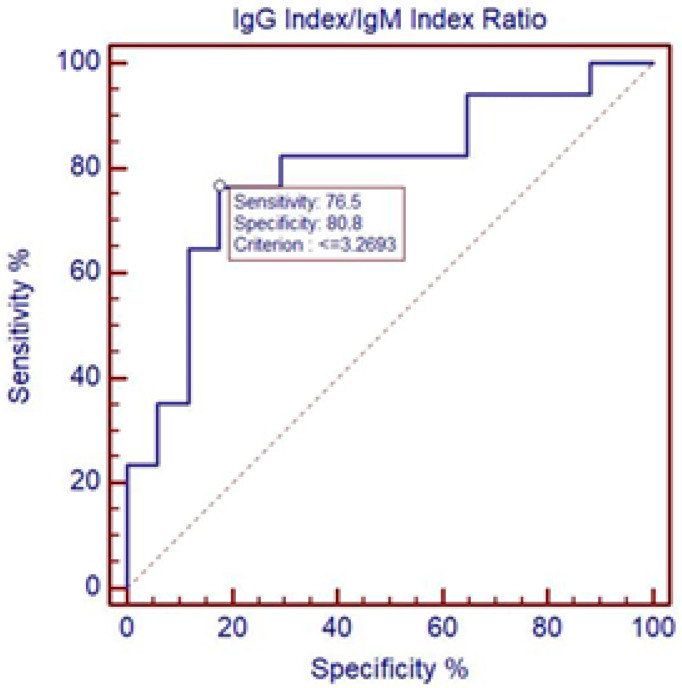
Differentiation between severe and mild course of TBE using IgG/IgM Index. ROC curve analysis showing that IgG/IgM Index differentiates between severe and mild courses of TBE at the cut-off 3.2693; specificity was 80.8% and sensitivity was 76.5%. AUC = 0.792, *p* < 0.05. CI (AUC) = 0.619 to 0.912.

ROC curve analysis showed that the IgG/IgM Index differentiates severe and mild courses of TBE at the cut-off of 3.2693; specificity was 80.8% and sensitivity was 76.5%, with AUC = 0.792, *p* < 0.05 (Figure 2).

No differences in antibody indexes between patients discharged with sequelae and patients with full recovery were observed.

The analysis of correlation showed a statistical significance between IgM Index and pleocytosis and protein concentration in CSF (r = −0.33 and r = −0.34, respectively) in the whole group of patients. These correlations were stronger in the meningitis group (IgM Index vs. pleocytosis r = −0.47 and vs. protein concentration r = −0.73), while in the meningoencephalitis group, no significant correlations were observed.

## 3. Discussion

Acute tick-borne encephalitis (TBE) has been the subject of extensive research, and its clinical presentation is well-documented, particularly in cases caused by the European subtype of the TBE virus (TBEV). The disease typically progresses in two distinct phases, observed in more than half of affected individuals. The first phase, often referred to as the early viremic phase, has an average duration of approximately 4 days, with a reported range of 1 to 8 days. This phase is characterized by non-specific, flu-like symptoms, including mild fever, fatigue, headache, and generalized muscle pain. Due to the non-specific nature of these symptoms, the early phase is frequently mistaken for a common viral infection, which may delay further diagnostic investigation.

Following the initial phase, there is usually a transient period of improvement, or even complete asymptomatic recovery, lasting about one week. However, in a significant proportion of patients, the disease progresses to a second, more severe phase, which is marked by neurological involvement. This phase manifests as various clinical forms of central nervous system (CNS) inflammation, including meningitis, meningoencephalitis, and, less commonly, meningoencephalomyelitis. Specifically, meningitis occurs in approximately 50% of adult patients, and meningoencephalitis in 40%, while meningoencephalomyelitis, the most severe and rare form, is observed in only 5–10% of cases. The second phase is associated with a wide spectrum of neurological symptoms, ranging from headache, photophobia, and neck stiffness in meningitis, to altered consciousness, seizures, or focal neurological deficits in meningoencephalitis or meningoencephalomyelitis.

There is no causative treatment for TBE. Therefore, vaccination remains the only effective means of preventing a severe disease course and the potential development of sequelae.

In the current database, information on long-term sequelae development was not available. At the time of discharge from hospital, 17 patients presented with various neurological or subjective symptoms (mostly cerebellar syndrome).

However, in our previous work, focused on long-term sequelae, we observed that 20.6% of patients may develop sequelae (mostly subjective). Patients with meningoencephalomyelitis were predisposed to neurological complications, while subjective symptoms were more commonly associated with meningoencephalitis [17,18].

Sodero et al. reported that children with encephalitis of various origins developed endocrine sequelae. This aligns with our previous findings concerning TBE, in which we observed that 16.9% of patients with severe TBE developed SIADH (syndrome of inappropriate antidiuretic hormone secretion) [19].

Independent risk factors for sequelae development included age and cerebrospinal fluid (CSF) protein concentration. A higher CSF protein concentration was associated with an increased risk of persistent late neurological complications [17,18]. Additionally, Siemieniako-Werszko et al. observed that patients who developed long-term sequelae presented with lower IgG intrathecal index at admission and higher IgG2/IgG1 index [8].

In comparison to the well-established understanding of the clinical course of TBE, much less is known about the factors that influence the severity of the disease. The available evidence remains inconsistent, and the mechanisms contributing to disease progression and outcome are still poorly understood. Our study adds to the growing body of research by highlighting the potential utility of serological markers, specifically the TBE IgM Index and the TBE IgG/IgM Index, as tools for differentiating between severe and mild courses of TBE. These indices may serve as valuable indicators in clinical practice, aiding in the early identification of patients at higher risk of severe neurological complications, and thereby facilitating more targeted monitoring and management strategies [9,20].

In addition to the observations outlined in the first paragraph of the discussion, the manifestation of severe tick-borne encephalitis (TBE) is associated with a complex interplay of clinical and laboratory indicators. Age emerges as one of the most significant factors influencing disease severity, with older individuals demonstrating a markedly higher risk of severe outcomes, as highlighted in several studies [21,22,23,24,25]. This correlation is likely due to age-related changes in the immune system, such as immunosenescence, which may impair the body’s ability to effectively respond to viral infections.

Beyond age, other demographic and health-related factors have been linked to more severe forms of TBE. For instance, male sex has been identified as a risk factor for increased disease severity, with men showing a higher likelihood of experiencing severe neurological complications [25]. Additionally, underlying comorbidities such as diabetes further exacerbate the risk, potentially due to the systemic inflammation and vascular damage associated with the condition [25]. In the analyzed cohort, no significant differences in disease severity were observed between male and female patients. This finding suggests that sex does not appear to influence the clinical presentation or severity of the disease in this population.

The clinical manifestation of TBE itself may also influence its severity. Patients with a monophasic presentation of the disease, characterized by the absence of the typical biphasic progression, are more likely to experience severe symptoms. This could reflect a more aggressive or rapid course of viral activity, as noted in previous research [22].

Interestingly, certain co-infections and prior immunological exposures appear to modify the clinical trajectory of TBE. For example, the concurrent presence of Lyme disease, caused by Borrelia burgdorferi, has been associated with a heightened risk of severe outcomes, possibly due to the compounded immune response triggered by both infections [21]. Moreover, earlier vaccination against TBE, while generally protective, has been paradoxically associated with severe illness in rare cases. This phenomenon may be attributable to incomplete vaccination schedules or waning immunity over time, emphasizing the importance of maintaining booster doses for long-term protection [26].

These findings collectively highlight the multifaceted nature of TBE severity, influenced by a combination of patient characteristics, comorbid conditions, and immunological factors. Understanding these correlations is essential for identifying individuals at higher risk, tailoring treatment strategies, and improving outcomes in severe cases of TBE.

Furthermore, Kaiser [23,27] has established that an elevated concentration of protein in the cerebrospinal fluid (CSF) is a significant prognostic marker for a more severe acute manifestation of tick-borne encephalitis (TBE); however, other studies have presented findings that challenge this perspective. Specifically, the study conducted by Günther et al. [28] introduced a contradictory viewpoint, indicating that low levels of early specific CSF IgM antibodies are associated with the development of more severe encephalitic symptoms in TBE patients. This observation diverges from the findings of our current study and adds complexity to the understanding of prognostic markers in TBE.

Günther et al.’s findings suggest that the early immune response, as measured by IgM antibody production within the CSF, may play a pivotal role in influencing the severity of the disease. Lower levels of these antibodies could imply a delayed or insufficient initial immune reaction, potentially allowing for greater viral replication and more extensive damage to the central nervous system (CNS).

The contrasting results from Günther et al. and our study emphasize the need for further research to clarify the relationship between immune response markers, such as IgM and protein concentration in CSF, and the clinical severity of TBE. A better understanding of these dynamics could lead to improved prognostic tools and more effective management strategies for patients with this complex and variable disease.

It is widely recognized that IgM antibodies against the tick-borne encephalitis virus (TBEV) play a key role in the immune response as the disease advances into its second, neurological stage. This stage is typically marked by the onset of more severe symptoms, including fever, headache, photophobia, and neurological deficits such as confusion, motor impairments, or seizures. The production of IgM antibodies is triggered during this phase as part of the body’s effort to combat the viral infection within the central nervous system (CNS).

The concentration of these antibodies peaks shortly after the onset of neurological symptoms and remains elevated for a period of approximately six weeks. This temporal pattern reflects the body’s attempt to mount an effective immune response against the virus, and it aligns with the progression of clinical symptoms. Importantly, this pattern of IgM production has significant clinical implications. By the time most patients present to the hospital, specific IgM antibodies are detectable in their blood samples. This makes IgM detection a highly reliable and timely diagnostic marker for confirming TBE in suspected cases.

The most probable link between intrathecal IgM production and the severity of TBE may be related to delayed seroconversion to IgG, which consequently results in an inadequate immune response to the virus. We have previously observed that delayed IgG antibody production correlates with the development of sequelae [8]. Furthermore, Bogovič et al. [29] reported that low levels of TBE virus-specific IgG antibodies in serum were associated with more severe manifestations of TBE.

Furthermore, the presence of IgM antibodies not only aids in the diagnosis of TBE but also provides valuable insights into the immune dynamics underlying the disease. The early appearance and persistence of IgM antibodies indicate an acute immune response aimed at neutralizing the virus. However, the level of these antibodies and their relationship to disease severity are areas of ongoing investigation. The detection of IgM antibodies is particularly useful in differentiating TBE from other viral CNS infections, which may have overlapping clinical presentations but distinct immunological profiles.

These findings underscore the critical role of serological testing in the diagnostic process, especially when combined with clinical assessment and cerebrospinal fluid analysis. By providing a window into the body’s immune response, IgM detection not only facilitates accurate and early diagnosis but also enhances our understanding of the pathophysiology of TBE and its progression. This knowledge is crucial for optimizing patient management and guiding future research into the immunological mechanisms of the disease [28,30].

In addition, data from 717 TBE individuals in Central Europe were analyzed by Bogovič et al., who discovered a negative correlation between the level of specific TBEV serum IgG and the severity of acute disease [29,31]. Radzišauskienė et al. claimed that delayed IgG intrathecal synthesis in TBE is connected to a more severe course of the illness [31]. However, in Siemieniako-Werszko et al.’s study, the intrathecal synthesis did not correlate directly with the clinical course of the illness [13]. Interestingly, previous studies did not concentrate on the usefulness of the TBE IgM Index and the TBE IgG/IgM Index in the assessment of the severity of the TBE illness—this aspect distinguishes our research from others. In our study, significantly lower IgG/IgM Index was associated with a more severe course of TBE. This imbalance between IgM and IgG production might be explained by either delayed IgG production or increased IgM production. As the rest of the results indicate (no statistical difference between IgG indexes between groups and higher IgM Index in severe TBE), the main reason is the overt IgM production in severe TBE.

In our study, we observed negative correlations between pleocytosis and protein concentration and IgM synthesis index, which suggest that IgM antibodies may lower the inflammatory response during TBE. However, these correlations were only observed in the meningitis group, and not in the more severe clinical form—meningoencephalitis.

Therefore, we can assume that although in meningitis, IgM effectively plays its protective role to the CNS, in meningoencephalitis, this beneficial effect is somehow limited.

The main limitation of our study is the small sample size and its restriction to a single-country setting, which may affect the generalizability of the results. Also, in this study we did not have data concerning long-term sequelae development. Therefore, multicenter studies with larger cohorts are necessary to validate our findings and provide broader insights. Additionally, the study is based on the IgG Index, IgM Index, and IgG/IgM Index, as was planned in previous studies to assess the severity of the course of TBE.

## 4. Conclusions

In this study, the IgM Index and the IgG Index/IgM Index ratio emerged as potential biomarkers for predicting the severity of TBE, providing valuable insights for clinical assessment. The findings suggest that delayed seroconversion to IgG may result in prolonged intrathecal IgM production, contributing to an inadequate immune response and increased disease severity. These observations underline the importance of timely immune response in managing TBE and highlight the potential utility of immunological indices in prognostic evaluation. Nonetheless, further research is warranted to elucidate the precise mechanisms underlying these associations and to validate these biomarkers in larger and more diverse cohorts.

## Figures and Tables

**Table 1:** Patient complaints at admission.

Symptoms at Admission *n* = 36		
	*n*	%
fever	36	100.0
headache	36	100.0
fatigue	13	36.1
nausea/vomiting	19	52.8
cerebellar syndrome	12	33.3
paresthesia	2	5.6
tremor	3	8.3
paresis	2	5.6

**Table 2:** Results of basic laboratory examinations.

Meningitis *n* = 18				Meningoencephalitis *n* = 18			
	Median	Min	Max	Median	Min	Max	*p*
CRP	5.7	0	21.7	5	0.6	79.5	1
WBC	10.7	4.8	15	9.9	6	14.5	0.77
pleocytosis	126.5	37	491	71.5	20	337	0.07
CSF protein concentration	71.5	42	133	73.5	43	117	0.86
CSF albumin concentration	46.9	23.4	93.5	52.2	23.5	81.8	0.84

## Data Availability

The data are available upon request to the corresponding author. The data are not publicly available due to privacy rules.
